# Raman Multi-Omic Snapshots of Koshihikari Rice Kernels Reveal Important Molecular Diversities with Potential Benefits in Healthcare

**DOI:** 10.3390/foods12203771

**Published:** 2023-10-13

**Authors:** Giuseppe Pezzotti, Yusuke Tsubota, Wenliang Zhu, Elia Marin, Takehiro Masumura, Takuya Kobayashi, Tetsuya Nakazaki

**Affiliations:** 1 Ceramic Physics Laboratory, Kyoto Institute of Technology, Sakyo-ku, Matsugasaki, Kyoto 606-8585, Japan; keeper.0925@gmail.com (Y.T.); wlzhu@kit.ac.jp (W.Z.);; 2 Department of Molecular Genetics, Institute of Biomedical Science, Kansai Medical University, 2-5-1 Shinmachi, Hirakata, Osaka 573-1010, Japan; 3 Department of Immunology, Graduate School of Medical Science, Kyoto Prefectural University of Medicine, Kamigyo-ku, 465 Kajii-cho, Kyoto 602-8566, Japan; 4 Department of Dental Medicine, Graduate School of Medical Science, Kyoto Prefectural University of Medicine, Kamigyo-ku, Kyoto 602-8566, Japan; 5 Department of Orthopedic Surgery, Tokyo Medical University, 6-7-1 Nishi-Shinjuku, Shinjuku-ku, Tokyo 160-0023, Japan; 6 Department of Applied Science and Technology, Politecnico di Torino, Corso Duca degli Abruzzi 24, 10129 Torino, Italy; 7 Department of Molecular Science and Nanosystems, Ca’ Foscari University of Venice, Via Torino 155, 30172 Venice, Italy; 8 Laboratory of Genetic Engineering, Kyoto Prefectural University, 1-5 Shimogamohangi-cho, Sakyo-ku, Kyoto 606-8522, Japan; masumura@kpu.ac.jp; 9 Department of Medical Chemistry, Kansai Medical University, 2-5-1 Shinmachi, Osaka Prefecture, Hirakata 573-1010, Japan; kobayatk@hirakata.kmu.ac.jp; 10 Experimental Farm, Graduate School of Agriculture, Kyoto University, Kizugawa 619-0218, Japan; nakazaki.tetsuya.4m@kyoto-u.ac.jp

**Keywords:** Raman spectroscopy, rice kernels, metabolites, molecular structure, statistical distributions

## Abstract

This study exploits quantitative algorithms of Raman spectroscopy to assess, at the molecular scale, the nutritional quality of individual kernels of the Japanese short-grain rice cultivar Koshihikari in terms of amylose-to-amylopectin ratio, fractions of phenylalanine and tryptophan aromatic amino acid residues, protein-to-carbohydrate ratio, and fractions of protein secondary structures. Statistical assessments on a large number of rice kernels reveal wide distributions of the above nutritional parameters over nominally homogeneous kernel batches. This demonstrates that genetic classifications cannot catch omic fluctuations, which are strongly influenced by a number of extrinsic factors, including the location of individual grass plants within the same rice field and the level of kernel maturation. The possibility of collecting nearly real-time Raman “multi-omic snapshots” of individual rice kernels allows for the automatic (low-cost) differentiation of groups of kernels with restricted nutritional characteristics that could be used in the formulation of functional foods for specific diseases and in positively modulating the intestinal microbiota for protection against bacterial infection and cancer prevention.

## 1. Introduction

The nutritional value and health-promoting properties of rice kernels are directly linked to their metabolomic, proteomic, glycomic, and lipidomic profiles, which, in turn, depend on genetic factors, environmental fluctuations, post-harvesting processing, and storage conditions [1,2,3]. Nowadays, gene-based markers have allowed for the multiplication of rice varieties through the synthesis of on-demand hybrids whose selectively accumulated gene alleles directly impact on specific nutritional traits, grain quality, and dietetic factors [4]. The so-called “genomic approach” includes tailoring specific grain proteins and amino acids [5,6], enriching with vitamins and specific minerals [7], controlling glycemic impact [8], and boosting antioxidant characteristics through enhanced fractions of phenolic and flavonoid compounds [9]. However, significant molecular diversity can be recorded within the same genomic species due to extrinsic factors, which impact on nutritional profiles [10]. This circumstance calls for an enhanced accessibility to real-time information based on a “multiomic approach”, which could enable consumers and healthcare operators to make cognizant and objective choices, irrespective of their heterogeneous criteria of selection.

In previous papers [10,11,12], the present authors illustrated the usefulness and flexibility of Raman spectroscopy in non-destructive and nearly real-time multi-omic analyses of renowned Japanese and international rice brands. The Raman spectrum represents a vibrational signature of molecules and a structurally sensitive “fingerprint” representative of structural details at the molecular scale, including types of chemical bonds and molecular symmetry. Modern Raman instruments are capable of detecting a Raman spectrum with a high efficiency and of recording it in nearly real time without sample manipulation or any need for extrinsic labeling procedures. The Raman spectrum concurrently encrypts a large amount of structural information at the molecular scale, which can be unfolded to a quantitative level once specific calibrations with basic compounds are performed. Our previous works [10,11,12] established and validated (upon comparison with standardized methods) a set of quantitative Raman algorithms for the determination of amylopectin and amylose concentrations, fractions of phenolic compounds, grain protein content, and secondary structures. The above Raman “quality” assessments of rice kernels are directly linked to the glycemic, antioxidative, nutritional, and digestibility characteristics of rice samples, respectively. Our previous studies applied the Raman method to ten different Japanese rice samples, four different Japanese glutinous paddy rice (mochigome) samples, and seven international brands specifically selected to cover a wide spectrum of structural characteristics. The data revealed significant diversity not only among different rice brands, but also within nominally “homogeneous” samples. Such a variety of structural characteristics has recently been widely paid attention to in crop science [13]. Several studies [14,15,16,17] have tracked down the origin of finer-level genetic diversity among domesticated rice species, including breeding systems, environmental effects, and the complexity of the breeding practices exercised by humans. With increasing the availability of rice genome sequences capable of resolving finer genetic structures in full, the interpretation of evolutionary relationships among rice groups/sub-groups and their link to geographical characteristics appear as nowadays feasible objectives. In synergy with refined genomic approaches, Raman spectroscopy could provide complementary knowledge linking multi-omic fluctuations to environmental effects and breeding practices.

In this paper, we will take a step forward and further exploit Raman technology to non-destructively characterize the glycomic, metabolomic, and proteomic inhomogeneities of rice; a statistical approach is applied here, which aims at characterizing a large number of rice kernels, both genetically and geographically homogeneous, with respect to their amylopectin-to-amylose and protein-to-carbohydrate ratios, fractions of phenylalanine and tryptophan aromatic amino acid residues, and fractions of protein secondary structures. Statistical distributions are built up through an analysis of individual Koshihikari rice kernels taken from exactly the same geographical area. Statistical distributions from different prefectures in Japan are also compared. Besides the relevance of regional differences within the same Japanese short-grain rice cultivar Koshihikari (already discussed in previous papers) [10,11], the stunning output of the present statistical analyses resides in the scattering breadth observed for all the metabolomic, proteomic, and glycomic parameters when comparing individual kernels harvested from exactly the same area.

The newly collected statistical results appear to encourage the practical development of appropriate Raman hardware solutions capable of automatically screening individual rice kernels in nearly real time for key applications in healthcare. Raman multi-omic snapshots could allow grouping rice kernels with strictly controlled characteristics and could provide unprecedented opportunities for exploiting low-cost/high-precision dietary therapies for multifactorial diseases, such as diabetes and chronic kidney diseases.

## 2. Experimental Procedures

### 2.1. Rice Samples

**Figure 1:**
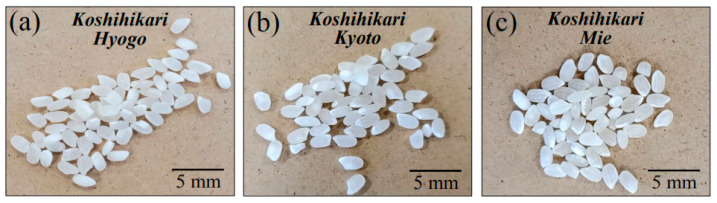
Photographs of the three investigated rice kernels of Koshihikari rice cultivar harvested in different Japanese Prefectures: (**a**) Hyogo, (**b**) Kyoto, and (**c**) Mie.

Three different samples of white (polished) rice cultivar Koshihikari, harvested in different regions of Japan (i.e., Hyogo, Kyoto, and Mie Prefectures in Figure 1a–c, respectively), were analyzed. The Koshihikari rice cultivar, developed in 1956, has been the most widely grown type of rice in Japan for more than 35 years. [18] The widespread popularity of the Koshihikari cultivar in Japan resides in its superior eating quality; more specifically, its sticky and chewy textures are its most pivotal features. According to standardized parameters, including appearance, flavor, taste, stickiness, and texture, the expert-panel judgment was at the highest rank (Toku A) for the Koshihikari Hyogo sample, at the second-highest rank (A) for the Koshihikari Kyoto sample, and at the third-highest rank (A’) for the Koshihikari Mie sample. All three investigated rice samples were harvested in the year 2021 and subjected to Raman spectroscopic analyses with no manipulation.

### 2.2. Raman Equipment

The Raman equipment used in this study included a triple monochromator (T-64000, Horiba/Jobin-Yvon, Kyoto, Japan) equipped with a multichannel liquid-nitrogen-cooled CCD, a confocal pinhole, and polarization filters. A high level of laser stray-light rejection, as compared to standard notch filter configurations, represents one of the key features of this equipment. The equipment included a holographic diffraction grating of 1800 gr/mm; the dispersion at a wavelength of 600 nm with the lattice 1800 gr/mm in subtraction mode was 0.64 nm/mm, while in addition mode, it was 0.21 nm/mm. The concurrent collection of a standard neon signal made it possible to achieve a high spectral resolution (better than 0.15 cm^−1^) and, thus, to accurately detect the position of Raman bands. The neon signal, external to the sample, was recorded at each spectral measurement and served as an internal calibration source against local fluctuations of the spectrometer apparatus (i.e., due to temperature or electrical noise).

The excitation source used in the present experiments was the 514.5 nm green line of an Ar-ion laser. Confocal experiments were conducted with an objective lens of ×100 magnification and a numerical aperture *NA* = 0.7; the pinhole aperture of the optical circuit was set to 100 μm. The confocal measurement conditions were: a laser power of 100 mW applied for 20 s through two or three successive (cumulative) acquisitions. Non-confocal experiments were conducted with a 20× optical lens and full pinhole aperture; the maximum laser power focused on the sample area was 35 mW applied for 20 s. These conditions enabled avoiding sample structural alterations arising from the laser irradiation and, in particular, preserving the pristine crystallographic structure of the samples owing to a controlled local heating of the irradiated zone.

### 2.3. Data Treatments, Machine Learning Approach, and Statistics

Spectral Raman lines were analyzed using a commercially available software package (LabSpec v4.02, Horiba/Jobin-Yvon, Kyoto, Japan). Spectral fitting was performed according to pseudo-Voigtian (i.e., mixed Gaussian/Lorentzian) functions after systematically subtracting a linear baseline. Except for the spectral fitting, all computational procedures were carried out with the aid of commercially available computational software (MATHEMATICA v7.0, Wolfram Research, Inc., Champaign, IL, USA).

Raman spectra were first treated with a baseline subtraction procedure and then automatically deconvoluted into a series of Gaussian–Lorentzian sub-bands. The baseline subtraction and deconvolution procedures were performed using the options available in the above-mentioned commercial software. The software applied a polynomial-fitting criterion for the baseline subtraction. All the spectra were analyzed for their relative intensity after normalization to selected signals. The spectral fitting was performed by the means of a customized automatic solver, which exploited a linear polynomial expression of the Gaussian–Lorentzian functions coupled with a machine learning working algorithm. This procedure enabled selecting the statistically most probable series of Gaussian–Lorentzian sub-bands, which fitted the experimental spectrum from the deconvoluted reference spectra of pre-selected compounds included in a library of key molecules, as previously described in detail [19,20]. Briefly, the computational procedure preserved the relative intensities, spectral positions, and full-width at half-maximum values for the individual sub-bands of the deconvoluted spectra from each elementary compound (i.e., within a scatter of ±3 cm^−1^, considering the possibility of slight alterations of the molecular structure). The above criteria provided the required constraints to univocally deconvolute the experimental spectra. The above-described computational program allowed an automatic spectral deconvolution, while also indicating sub-bands whose signal intensity was largely contributed by a single reference molecule (>90%).

The statistical relevance of the experimental data was analyzed by computing mean values and standard deviations. Their statistical validity was evaluated by applying a one-way analysis of variance (ANOVA) [21]. Within each sample size, a significance level of 0.05 (reported in the graphs as *p*-value < 0.05) was considered to be statistically significant and labeled with two asterisks.

A Post Hoc Tukey’s Honest Significant Difference (HSD) test was performed to determine which groups were significantly different from each other and the results are expressed as critical values (*Q*), as determined by the formula:

(1) $$Q=\frac{\bar{X_{i}}-\bar{X_{j}}}{\sqrt{\frac{MSE}{n}}}$$

where $\;\bar{X_{i}}$ and $\bar{X_{j}}$ are the means of the two groups being compared, MSE is the mean squared error from the ANOVA, and n is the number of observations in each group (n = 300). When the calculated *Q* statistic exceeded the critical value from the distribution, we assumed a statistically significant difference between the means of the two groups being compared.

## 3. Experimental Results

### 3.1. The Raman Spectrum of Rice and Related Quantitative Algorithms

**Figure 2:**
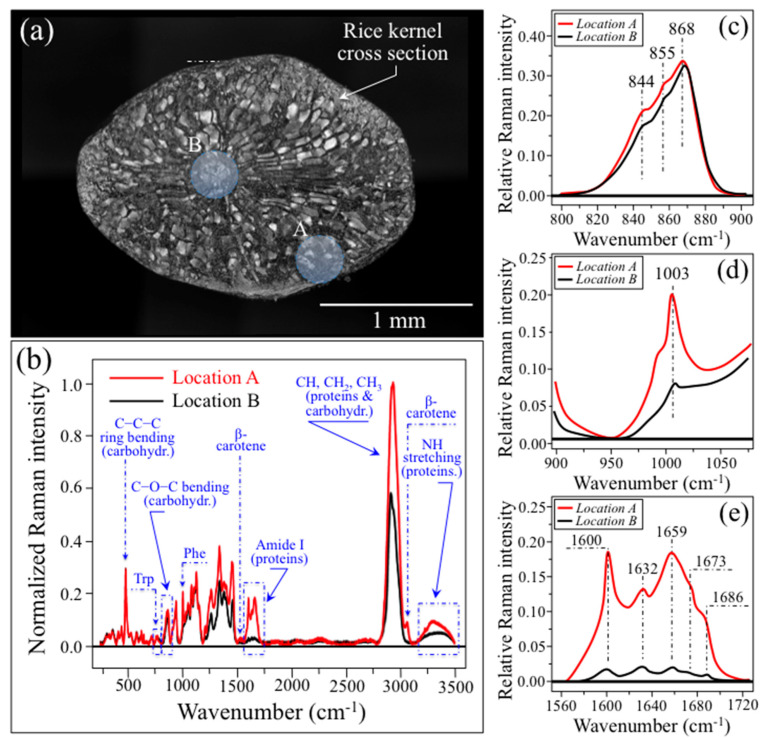
(**a**) Cross-section of a Koshihikari Kyoto rice kernel: a peripheral location A and a central location B are marked on the kernel; (**b**) Raman spectra collected at locations A and B in (**a**) after spectral intensity normalization to the glucose ring vibration band at 478 cm^−1^ (labels in inset explained in text). Three specific spectral features are enlarged and shown with higher spectral resolution: the region 800~900 cm^−1^ corresponding to C–O–C bending in carbohydrates (**c**), the phenylalanine band at around 1003 cm^−1^ (**d**), and the Amide I signal from proteins (between 1600 and 1700 cm^−1^) (**e**), as detected at locations A than in B. The labels given in inset to each spectral region represent the spectral locations of the observed maxima in each zone (in cm^−1^ units).

Preliminary cross-section experiments were specifically designed to locate differences in metabolite contents between the core and the shell of individual kernels. Figure 2a shows the cross-section of a Koshihikari Kyoto rice kernel. Cutting was performed with a hand-held diamond-coated surgical blade to obtain a good surface finish with only limited plastic deformation. In the figure, two selected locations are marked for non-confocal laser spots in the peripheral and central zones of the cross-section (labeled as A and B, respectively). The Raman spectra collected at locations A and B are shown in Figure 2b. After normalization to the glucose ring vibration band at 478 cm^−1^, four main features could be observed from comparing the spectra from the two different locations: (i) slight morphological differences could be noticed in the region of 800~900 cm^−1^, which corresponds to C–O–C bending in carbohydrates; (ii) the phenylalanine band at around 1003 cm^−1^ was significantly stronger in the peripheral zone A than the central zone B; (iii) similar to (ii), the Amide I signal (between 1600 and 1700 cm^−1^) was ~7 times stronger in A than in B; and, (iv) the bands in the high-frequency region were generally stronger at location A, but also a new band appeared at ~3070 cm^−1^. The above items (i)~(iii) were detailed in their respective high-resolution spectral intervals, as given in Figure 2c–e, respectively.

The C–O–C bending zone (800~900 cm^−1^; Figure 2c) presented three distinct maxima (at 868, 855, and 844 cm^−1^) at relative intensities that slightly differed for zones A and B of the kernel cross-section. Signals in the C–O–C bending zone are expected to show variations as a consequence of differences in the type of C–O–C bond. The C1–O–C6 Raman signal at 844 cm^−1^ is typical of branching structures of the glucose ring and, therefore, only belongs to amylopectin. Conversely, both signals at 868 and 855 cm^−1^, related to C5-O-C1 (within ring) and C1-O-C4 (at chain linkage) bonds, respectively, belong to both amylose and amylopectin structures [22]. Therefore, a higher relative intensity for the maximum located at 844 cm^−1^, as detected in the peripheral zone A as compared to zone B, proves that the former zone is richer in terms of amylopectin structure as compared to the latter. As shown in the next section, upon exploiting this distinction among the signals from different C–O–C bonds, it is possible to set an algorithm that quantitatively locates the fractions of amylose and amylopectin from Raman spectroscopic assessments of rice kernels.

The sharp signal detected at ~1003 cm^−1^ (Figure 2d) is characteristic of benzene ring breathing in aromatic amino acid phenylalanine [23]. This signal is seen as a shoulder in the spectra at both locations A and B, but its relative intensity at the former location is more than twice that at the latter. A similar trend was found for the tryptophan Raman signal at ~768 cm^−1^ (labeled as Trp in Figure 2b). This signal arises from breathing vibrations of the indole ring [24], which represents a peculiar feature of the aromatic amino acid tryptophan. The signals at 1003 and 768 cm^−1^ can be used to quantitatively characterize the weight fractions of phenylalanine and tryptophan, respectively, in the rice kernels, provided that quantitative equations are built up through preliminary calibrations. Quantifications of aromatic amino acid weight fractions in individual rice kernels will be discussed in detail in the next section.

The spectral region in Figure 2e gives a comparative estimate of the protein content through the Raman relative intensity of the Amide I bands (representative of proteins). According to Sadat and Joye [25], the regions 1649~1660 cm^−1^ and 1660~1675 cm^−1^ can be attributed to α-helix and random coil structures, respectively. The regions at 1620~1647 cm^−1^ and 1665~1680 cm^−1^ both represent the β-sheet structure, while bands centered in the wavenumber interval 1680~1699 cm^−1^ are signature bands for β-turn structures. In the Amide I zone of a rice kernel, we located distinct maxima at 1600 (α-helix), 1632 (β-sheet), 1659 (β-sheet), 1673 (random coil), and 1686 (β-turn) cm^−1^. The relative intensity of these bands (representative of proteins) to the glucose ring stretching band at 478 cm^−1^ (representing the cumulative amount of carbohydrates) characterizes the protein-to-carbohydrate ratio of the kernel, while the ratio between α-helix and random coil band intensities relates to digestibility, and the more disordered the secondary structure of proteins, the higher its digestibility. The approach adopted here is similar to that proposed by Ren et al. [26], which was validated by the means of chemical analyses. However, those researchers used the ratio between the Amide III band at 1278 cm^−1^ and the carbohydrate band at 362 cm^−1^ (C–C stretching). We instead selected the stronger signal at 478 cm^−1^ as a reference signal for polysaccharides and the Amide I band (instead of the Amide III signal), because the Amide III signal intensity could also be contributed by lipids.

The high-frequency signals in the interval of 2800~3500 cm^−1^ were significantly stronger when the spectra were recorded in the peripheral location A as compared to the central location B of the kernel (Figure 2b). Signals in the high-wavenumber spectral zone might reflect the higher amount of proteins recorded in the Amide I spectral zone for the peripheral location (above). However, the strongest band in the overall spectrum, which is centered at ~2900 cm^−1^, mainly arises from C–H stretching in the glucose ring, but also contains contributions from C–H_2_ and C–H_3_ stretching in both proteins and carbohydrates. Conversely, the stronger N–H stretching signal observed for location A between 3200 and 3500 cm^−1^ is a clear indication of a higher protein content in the peripheral zone of the kernel, because N is not present in carbohydrate structures [27]. An additional feature in the spectrum of location A in Figure 2b is the sharp band at ~3070 cm^−1^. This band can be assigned to C–H stretching in the aromatic rings of the β-carotene structure [28,29]. The presence of this well-isolated C–H signal, which fits well with the other sharp feature observed at ~1525 cm^−1^ (C=C stretching) from β-carotene, can be considered as a spectroscopic fingerprint for carotenoids. The presence of β-carotene represents an important nutritional characteristic, because it is converted into vitamin A in the human body. Rice endosperm generally does not contain β-carotene unless specific precursors for phytoene synthase are incorporated [30].

The set of above data clearly show a high degree of inhomogeneity in the molecular structure within an individual rice kernel. This circumstance calls for an appropriate laser probe calibration in terms of penetration depth, if data representative for the entire kernels should be retrieved by the means of a single-shot Raman measurement.

### 3.2. Raman Probe Size and Probe Calibrations for Single-Shot Analyses

An assessment of the Raman probe was performed in order to prove the feasibility of a fast, non-destructive, on-site evaluation of the nutritional value of individual rice kernels. For this purpose, a set of Raman experiments was purposely designed to evaluate the feasibility of in-depth Raman analyses and the effect of probe size and spatial scattering response on the measured metabolite fractions.

**Figure 3:**
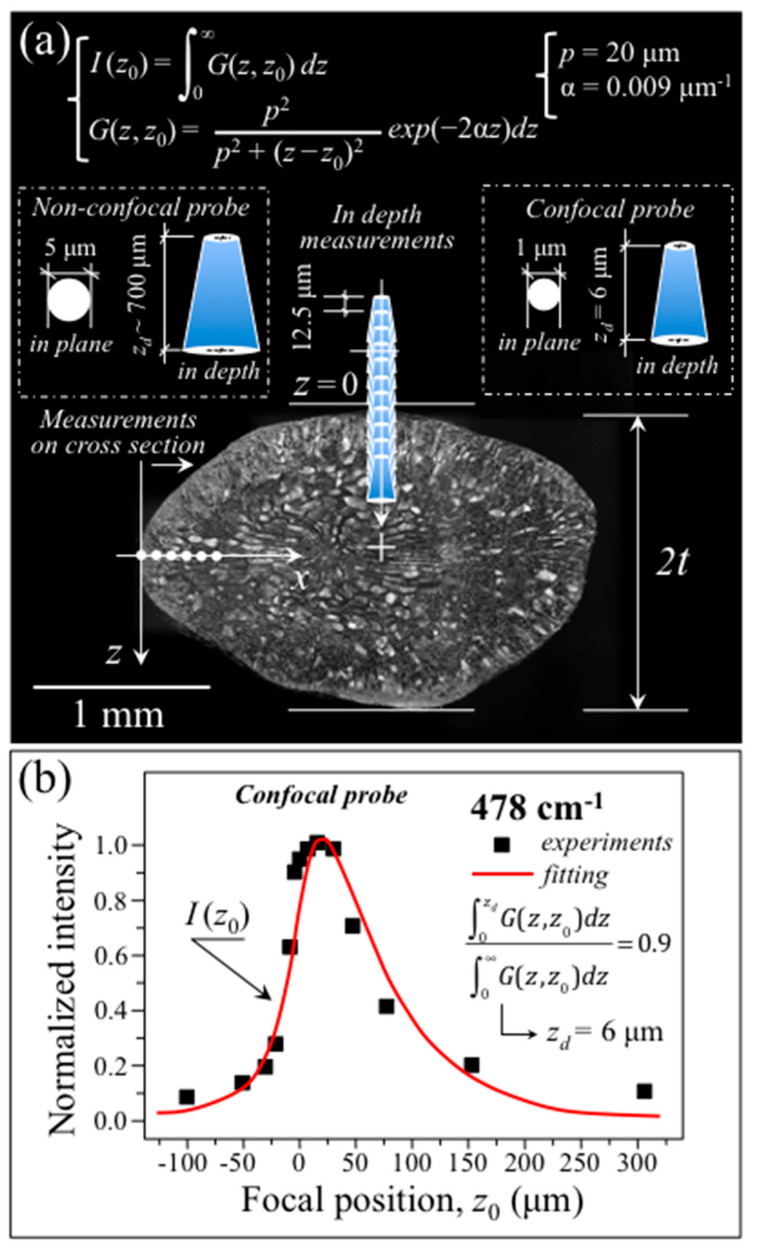
(**a**) Schematic drafts of 20× non-confocal and 100× confocal Raman probes with their estimated penetration depths (given in inset); two procedures of confocal probe calibration, consisting of in-depth non-destructive scanning along the *z*-axis with 12.5 μm steps and in-plane cross-sectional scanning along the *X*-axis with 50 μm steps are also schematically shown; and (**b**) results of confocal probe calibration given in terms of the 478 cm^−1^ Raman intensity distribution, *I*(*z_0_*), which is in turn expressed by means of the so-called probe response function, *G*(*z*,*z_0_*), [31] as defined by the equations shown in inset to (**a**). An estimate of the 100× confocal and 20× non-confocal probe depths contributing for 90% of the total Raman intensity observed were determined as *z_d_* = 6 μm and 700 μm, respectively (labels in inset to (**a**)), according to the equation shown in inset to (**b**) [31].

As a first step, the in-depth response of a 100× confocal probe (as described in Section 2.2) was determined according to a previously proposed procedure [31]. Figure 3a shows a schematic draft of the in-depth confocal probe calibration, which consisted of scanning along the *z*-axis (focal plane location, *z*_0_) with 12.5 μm steps while recovering the intensity dependence, *I*(*z*_0_), of the 478 cm^−1^ Raman band. The results of such a probe calibration are shown in Figure 3b. The retrieved intensity distribution as a function of the abscissa *z*_0_ can be modeled according to the concept of probe response function, *G*(*z*,*z*_0_), namely, the function describing the Raman intensity contribution from each depth, *z*, when the focal plane is set at *z*_0_. *G*(*z*,*z*_0_) is a function of the absorption coefficient, α, and of the probe response parameter, *p*, according to the equation shown in inset to Figure 3a [31]. The parameters *p* and α for the rice kernel were found equal to 20 μm and 0.009 μm^−1^, respectively, while the penetration depth contributing 90% of the total Raman intensity in a 100× confocal probe configuration could be estimated as *z_d_* = 6 μm, according to the equation shown in Figure 3b (also draft in inset to Figure 3a). A similar probe calibration procedure conducted on the 20× non-confocal probe gave a probe penetration depth at a 90% signal intensity equal to 700 μm (draft in inset to Figure 3a). Such a penetration depth roughly corresponds to 35~50% of the total kernel thickness, 2*t* (Figure 3a).

The above probe calibration experiments prove that the relatively high transparency to visible laser of the rice kernel might allow Raman measurements down to depths comparable to about half the kernel thickness.

**Figure 4:**
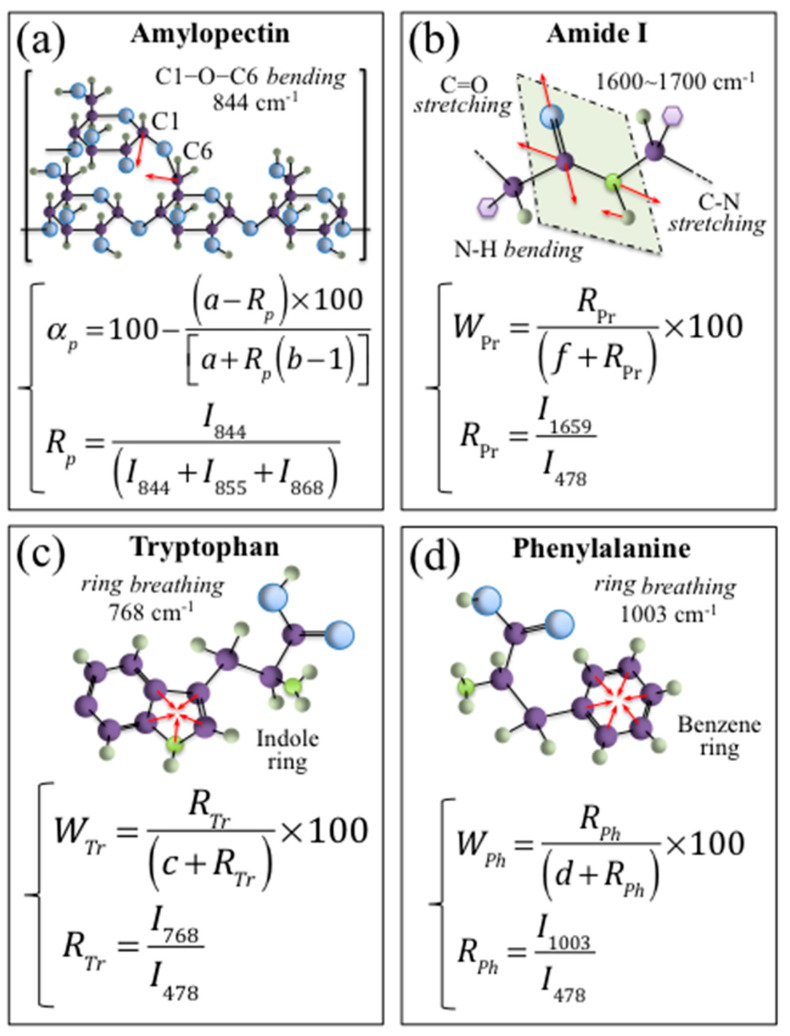
Selected vibrational modes and the Raman algorithms (developed in Refs. [10,11,12]) used for the quantitative determination of (**a**) amylopectin fraction in starch, *α_p_*, (**b**) weight fraction of proteins, *W_Pr_*, (**c**) weight fraction of tryptophan, *W_Tr_*, and (**d**) phenylalanine, *W_Ph_*, aromatic amino acid residues. Purple, green, grey and light blue spheres represent carbon, nitrogen, hydrogen, and oxygen, respectively. The red arrows represent the vibrational modes of the respective atomic bonds.

In previous papers [10,11,12], we established quantitative Raman algorithms to quantify the fraction of amylopectin, *α_p_*, the weight fraction of proteins, *W_Pr_*, and the weight fractions of tryptophan, *W_Tr_*, and phenylalanine, *W_Ph_*, in rice samples; we also validated them by the means of independent (standardized) methods, and applied them to different Japanese and international cultivars. Figure 4a–d summarizes the selected vibrational modes and the Raman algorithms developed for a quantitative determination of *α_p_*, *W_Pr_*, *W_Tr_*, and *W_Ph_*, respectively.

**Figure 5:**
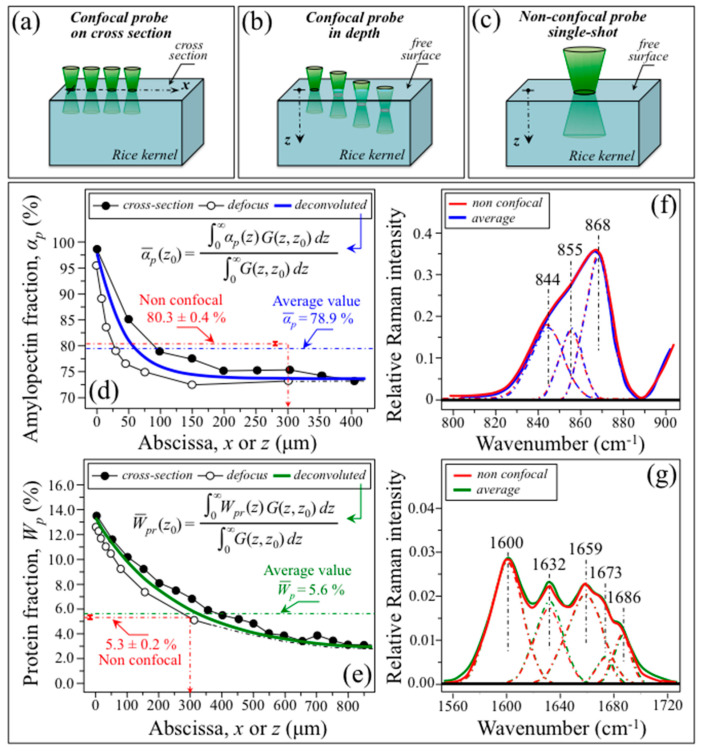
Procedures for collecting confocal (100×; pinhole aperture equal to 100 μm) Raman line-scans on (**a**) kernel cross-section (along the *x*-axis), and (**b**) non-destructively in-depth from kernel surface (along the *z*-axis); and (**c**) single-shot measurement in non-confocal probe configuration (20×; full pinhole aperture) and focus on the kernel surface. In (**d**,**e**), spatial distributions along kernel sub-surface as detected for amylopectin and protein fractions, respectively, for probe configurations given in (**a**,**b**). Probe deconvoluted distributions (full lines) were obtained by solving the integral equations in inset to the respective figures; [31] in inset, also a comparison between *α_p_* and *W_Pr_* values averaged over cross-section line-scan data (*x* = 0~*t*) and values obtained in one-shot Raman measurements with the non-confocal probe focused on the kernel surface (i.e., as shown in (**c**)). For both distributions, the one-shot value corresponds to values at a depth of ~300 μm. In (**f**,**g**), a comparison is given between the non-confocal one-shot spectrum (**c**) and the spectrum averaged over a line scan along the *X*-axis (*x* = 0~*t*) for the region 800~900 cm^−1^ (C–O–C bending in carbohydrates) and the Amide I region from proteins (between 1600 and 1700 cm^−1^), respectively. Spectra are deconvoluted into Gaussian–Lorentzian sub-bands (preserving the same color of the spectral lines), as explained in text.

As a successive step, Raman spectra were collected in a 100× confocal probe configuration (pinhole aperture equal to 100 µm) with line scans on the kernel cross-section along the *X*-axis (Figure 5a) and (non-destructively) in-depth from the free surface of the kernel along the *Z*-axis (Figure 5b). From the above two sets of measurements, the distributions of amylopectin fraction, *α_p_*, and protein weight fraction, *W_Pr_*, internal to the rice kernel were computed upon exploiting the quantitative algorithms given in Figure 4a,b, respectively, and compared. As an independent experiment, a single-shot non-destructive measurement was also recorded with a 20× non-confocal probe with full pinhole aperture (measurement conditions in Section 2.2) on the same kernel (Figure 5c). The *α_p_* and *W_Pr_* property distributions measured in confocal configuration by both cross-section and in-depth line-scans are compared in Figure 5d,e, respectively. As seen, both distributions presented high values at the kernel surface with steep monotonic decreases toward the kernel center. From these sets of confocal data, it appears that the penetration depth estimated for the 20× non-confocal probe focused on the kernel surface (i.e., 700 µm; draft in inset to Figure 3a), covering the kernel with enough depth to probe in full the zone of main nutritional characteristics. In other words, the presence of such steep gradients in nutritional components along the kernel depth is an important (and fortunate) characteristic, which opens up a path towards the fast, accurate, and non-destructive evaluation of kernel properties, as discussed later.

According to a data treatment procedure, the in-depth *α_p_* and *W_Pr_* distributions were deconvoluted to take into account the finite size of the confocal probe. This computational task was accomplished upon solving the integral equations shown in inset to Figure 5d,e, respectively (also the deconvolution procedure described in Ref. [31]). The deconvoluted distributions lay closer than those from the original in-depth scans to the respective *X*-axis distributions, only slighting underestimating the cross-sectional values in both *α_p_* and *W_Pr_* cases. These data prove that non-destructive in-depth Raman characterizations of metabolite profiles in rice kernels are possible to a degree of precision (i.e., errors < 3% and < 6% for *α_p_* and *W_Ph_*, respectively) by the means of a confocal Raman probe scan.

As a final step, a comparison was made between a single-shot spectrum collected with a 20x non-confocal probe focused on the kernel surface (Figure 5c) and a spectrum averaged over the set of spectra obtained upon the *x*-axis (cross-sectional) line-scan at *x* = 0~700 μm (Figure 5a). A comparison is shown in Figure 5f,g for the amylose/amylopectin (800~900 cm^−1^) and Amide I (1560~1720 cm^−1^) zones, respectively. The figures also show the respective spectral deconvolutions into sub-bands related to different C–O–C bonds and secondary protein structures, respectively, as described in Section 3.1. In both cases, the very close morphology of the non-confocal spectrum and the spectrum averaged from the cross-sectional scan was reflected in very similar values of amylopectin and protein fractions. Non-confocal and average *α_p_* and *W_Pr_* values are given in the inset to Figure 5d,e, respectively. Their differences were relatively small, namely 1.7 and 5.3%, in the cases of amylopectin and proteins fractions, respectively.

In summary, the full set of probe calibration data shows that the transparence and adsorption characteristics of rice kernels can enable non-destructive in-depth Raman analyses. Moreover, the relatively steep gradients of amylopectin and protein distributions from the kernel surface towards its center make it possible to obtain accurate property evaluations from the one-shot probing of individual kernels in a 20× non-confocal configuration with full pinhole aperture.

### 3.3. Statistical Distributions among Genetically Homogeneous Rice Kernels

**Figure 6:**
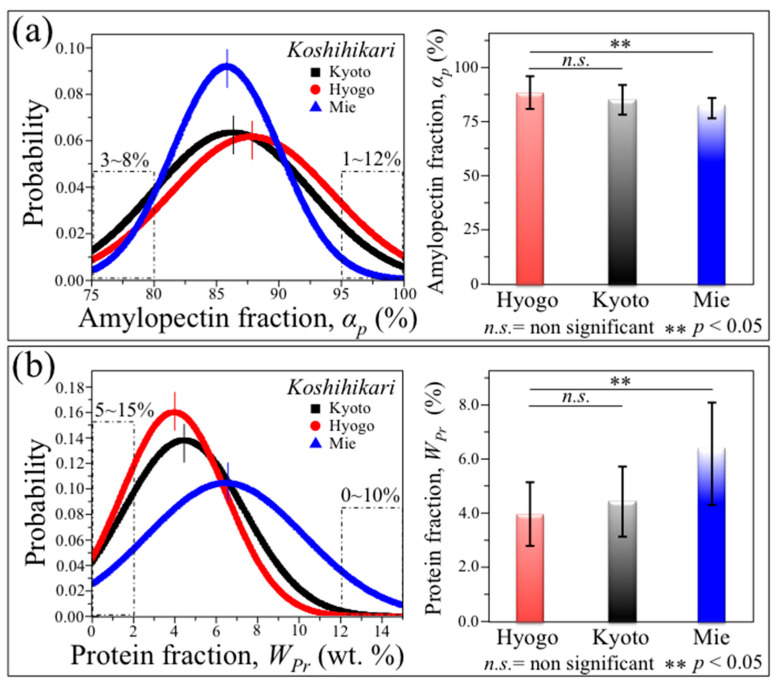
Results of statistical analyses performed on nutritional traits from 300 individual kernels for each regionally diversified Koshihikari (legends); statistical distributions recorded within the kernel populations are given for (**a**) amylopectin and (**b**) protein fractions (*α_p_* and *W_Pr_*, respectively). On the right side of each panel, average values for each distribution are plotted with the respective standard deviations and the related statistical validations according to ANOVA [21].

**Figure 7:**
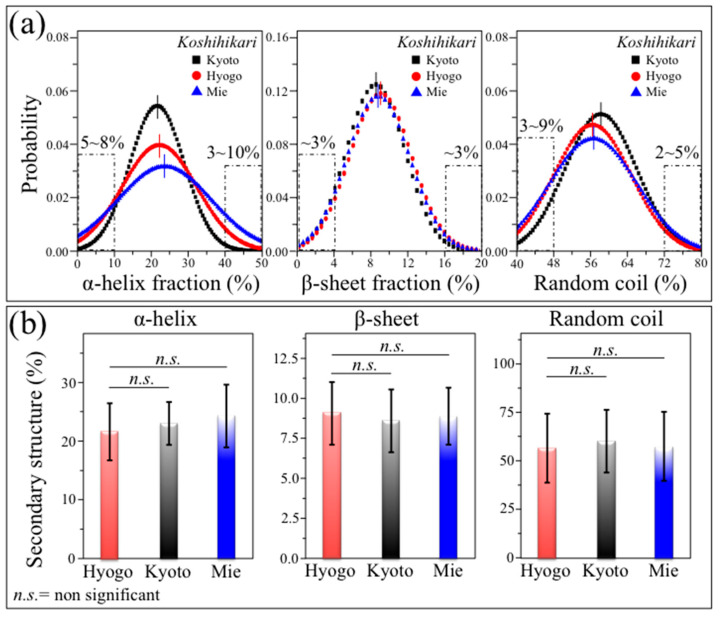
Results of statistical analyses performed on nutritional traits from 300 individual kernels for each regionally diversified Koshihikari (legends): (**a**) statistical distributions recorded within the kernel populations for the fractions of different protein secondary structures. In (**b**), the average value of each distribution is plotted together with its standard deviations; statistical validations are given according to the ANOVA [21].

**Figure 8:**
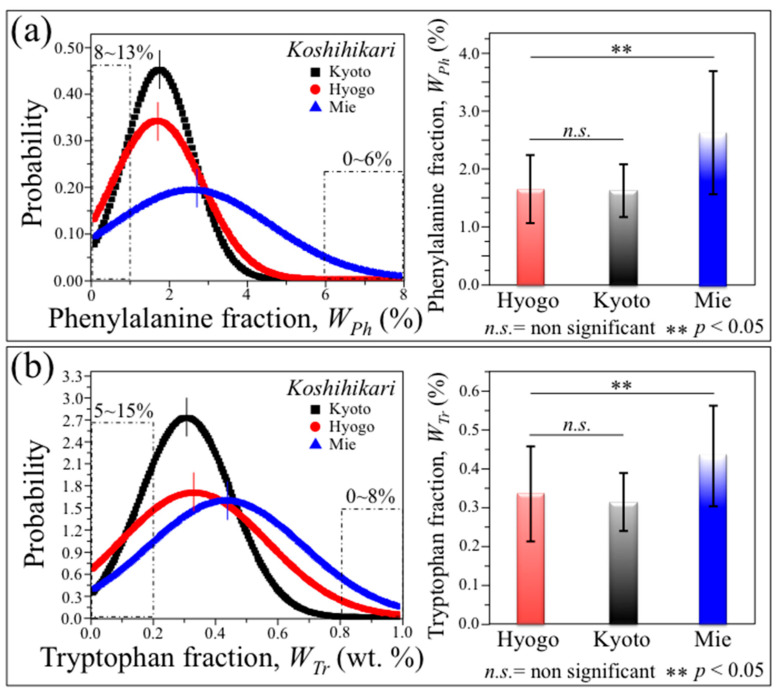
Results of statistical analyses performed on nutritional traits from 300 individual kernels for each regionally diversified Koshihikari (legends); statistical distributions recorded within the kernel populations are given for (**a**) phenylalanine and (**b**) tryptophan weight fractions. On the right side of each panel, average values are plotted for each distribution together with their standard deviations; the related statistical validations are given according to the ANOVA [21].

Figure 6, Figure 7 and Figure 8 show the results of the Raman statistical analyses performed on the nutritional traits from 300 individual kernels for each regionally diversified Koshihikari. Table 1 summarizes data proving the statistical validity of the collected data in terms of Q values, as calculated from Equation (1). In Figure 6a,b, the statistical distributions recorded among the individual rice kernels are given for amylopectin, *α_p_*, and protein fractions, *W_Ph_*, respectively. For each set of data, average values and standard deviations were plotted with related statistics (right side of each panel). From these sets of data, it emerges that the Koshihikari Mie is the highest in proteins and the lowest in amylopectin, which matches with the result of its (third-lowest) rank *A’*, as assigned by a standardized phenomenological analysis (Section 2.1). The Hyogo and Kyoto varieties showed closer statistical characteristics, with the former being the highest and lowest in amylopectin and proteins, respectively. The ranking Toku A (Hyogo) and A (Kyoto) for these two samples reflects a lower sensory judgment for protein-rich rice varieties, as previously reported by Nakayama [32]. As important characteristics, the statistics retrieved on individual kernels show that ~8% of a kernel population fraction in Koshihikari Kyoto possessed a starch composition with amylopectin content of <80% (values in inset to Figure 6a), while the Koshihikari Mie sample contained a protein weight fraction of >12% in a kernel population as high as 10% (values in inset to Figure 6b).

Remarkably, the high-protein content of the Koshihikari Mie sample is comprehensive of a population of ~10% of grains with an α-helix content of >40% (values in inset to Figure 7a). As a general trend, the kernels high in α-helix conspicuously corresponded to those low in random coil secondary structures, while the fraction of β-sheet showed a relatively sharp distribution, which remained conspicuously unchanged among the Koshihikari samples from different Japanese prefectures (plots in Figure 7b). These structural circumstances locate the investigated Koshihikari Mie sample as a suitable candidate from which a significant fraction of high-protein/low-digestibility kernels could be collected for dietetic use.

Regarding the statistical distributions and fractional contents of the aromatic amino acid residues phenylalanine (Figure 8a) and tryptophan (Figure 8b), again the investigated Koshihikari Mie sample was the richest one, while both the Hyogo and Kyoto brands showed ~1/3 lower fractions for both amino acids, with no statistically significant difference to each other. In the Koshihikari Mie sample, about 6% of the kernels contained >6 wt.% of phenylalanine (values in inset to Figure 8a), while ~8% of the kernels incorporated >0.8 wt.% of tryptophan (values in inset to Figure 8b). The majority of those aromatic residue-rich kernels were also the richest in proteins, thus suggesting that the hypothesis of harvesting kernels with specific nutritional properties from commercial rice samples is possible and their exploitation in tailoring rice nutritional quality is a realistic task.

**Figure 9:**
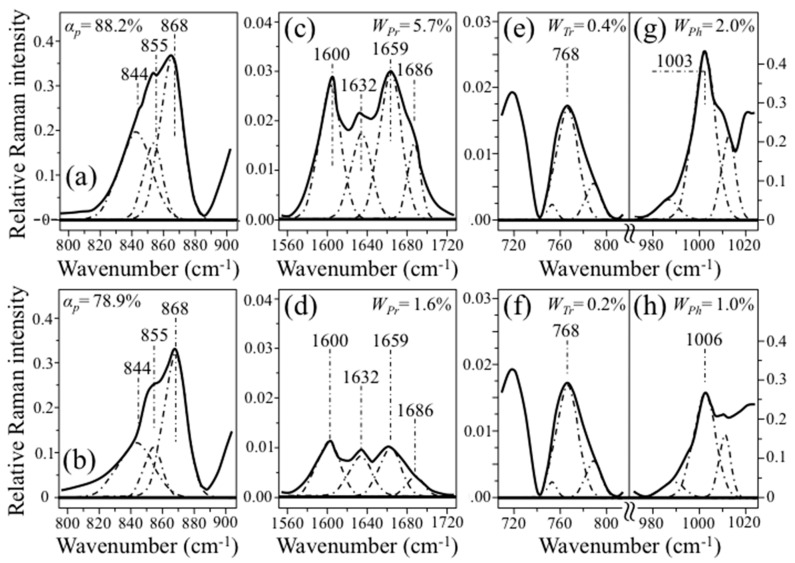
Spectral characteristics of one-shot monitored kernels from the Koshihikari Kyoto sample with the highest and the lowest *α_p_* ((**a**,**b**), respectively), *W_Pr_* ((**c**,**d**), respectively), *W_Tr_* ((**e**,**f**), respectively), and *W_Ph_* ((**g**,**h**), respectively). The spectral regions were deconvoluted as explained in Section 3.1.

Finally, it is worth noting that the spectral differences between kernels with different nutritional characteristics were quite bold for any of the nutritional parameters investigated. Figure 9 shows the spectral characteristics of one-shot monitored kernels from the Koshihikari Kyoto sample with the highest and the lowest *α_p_* (88.2 vs. 78.9%; in (a) and (b), respectively), *W_Pr_* (5.7 vs. 1.6%; in (c) and (d), respectively), *W_Tr_* (0.4 vs. 0.2%; in (e) and (f), respectively), and *W_Ph_* (2.0 vs. 1.0%; in (g) and (h), respectively). Thus, the detection of such clear spectral differences appears to be feasible with a non-destructive approach and within the time frame of a single-shot measurement.

## 4. Discussion

### 4.1. Feasibility of Real-Time Raman Multi-Omic Snapshot of Rice Kernels

The high potentiality of multi-omics in crop research is nowadays well recognized for its unique capacity to discover specific biomarkers and monitor crop yield and quality [33,34]. Crop multi-omics has, so far, been mainly pursued according to two analytical platforms: mass spectrometry and nuclear magnetic resonance coupled to liquid or gas chromatography [35]. These analytical methods have been quite successful in showing crop traceability and fine genetic variations in response to environmental and biotic/abiotic stresses. Accordingly, they have widely been used in both promoting crop quality and tailoring specific crop characteristics linked to geographical origin [13]. An additional method which has successfully been applied to quantitative crop multi-omics is Fourier-transform ion-cyclotron-resonance mass spectrometry [36]. This method, which is capable of simultaneously detecting thousands of metabolites with an extremely high resolution and accuracy, has so far mainly been employed to quantify fractions of bioactive nutraceutical compounds or to substantiate the extent to which crop quality has been compromised by the occurrence of pests and diseases in plantations [37,38,39]. Despite their quite successful outputs in molecular profiling approaches to plant phenotypic classifications and crop breeding strategies, the above spectroscopic approaches to quantitative crop multi-omics are time-consuming, destructive, and involve high costs, besides also requiring the use of expensive equipment. Accordingly, the application of any of them to the real-time/non-destructive screening of large (statistically meaningful) numbers of rice kernels (i.e., spanning from thousands to tens of thousand units per day in automatic mode) is both practically and economically unfeasible.

**Figure 10:**
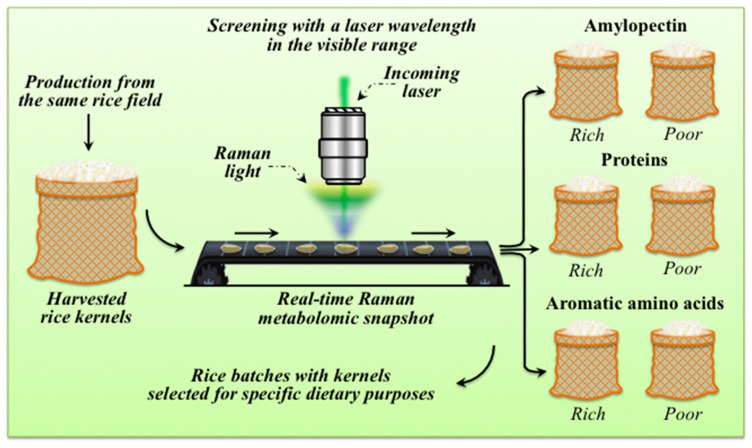
Schematic draft of an automatic procedure enabling individual rice kernels enriched in specific nutritional traits to be non-destructively screened on-line and automatically relocated into specific groups with targeted molecular characteristics.

The present study has unveiled, for the first time, the possibility of exploiting a real-time (targeted) Raman multi-omic approach for a realistically feasible, sufficiently precise, and statistically meaningful nutritional analysis of individual rice kernels. As discussed in detail in the next section, the present findings are important because single-kernel rice multi-omics has a huge potential in the field of healthcare nutrition. We demonstrated here that the scatters in nutritional traits among rice kernels in commercially available samples of the Koshihikari rice cultivar are wide. Accordingly, the search for kernel fractions with specific nutritional trends upon screening within commonly available rice batches with nominal (genetic and geographical) homogeneity is meaningful. Moreover, the statistically quantitative metabolite profiling of a large number of individual rice kernels becomes a feasible target through a Raman multi-omic-snapshot approach, since we proved that a conventional laser probe in the visible wavelength could penetrate a rice kernel to a depth enough for providing its average nutritional values at once. Thus, rice kernels enriched in specific nutritional traits could non-destructively be screened and automatically relocated into specific kernel groups with targeted molecular characteristics. Figure 10 gives a schematic draft of this newly proposed automatic procedure.

The currently available Raman analytical platforms have been employed to construct a number of different strategies for real-time (targeted) molecular fingerprinting in a variety of different fields [18,40,41,42]. Since it enables rapid and non-destructive measurements, the Raman technique is already routinely applied as a tool for quality control and on-line analyses in the pharmaceutical industry [43,44,45]. As shown in the present study, a non-confocal Raman probe configuration, which enables non-destructive in-depth screening without cross-sectioning, substantiates the technological feasibility of single-shot, non-confocal Raman measurements of individual rice kernels using a laser beam in the visible wavelength range. Therefore, similar to well-established Raman pharmaceutical analyses, which target the online continuous identification of different formulations and the polymorphic monitoring of active substances in individual tablets, Raman analyses can target selected metabolites and protein secondary structures in individual rice kernels. In an analogy with pharmaceutical quality control, the present paper proposes to apply the online Raman spectroscopic approach to rice kernel screening in order to customize their nutritional traits for healthcare purposes.

### 4.2. The Importance of Rice Kernel Multi-Omics in Modern Healthcare

The nutritional quality of rice depends on the amylose, amylopectin, protein, and amino acids contents of its grains. Characteristic fractions of these metabolites, which are synthesized and genetically regulated at the initial stage of kernel development, develop gradually with time through successive stages of grain maturation, drying, and dormancy [46,47]. All developmental stages involve substantial spatiotemporal metabolic rearrangements and generally obey gene expression programs [48]; however, environmental effects during the maturation stage play a fundamental role in the final kernel composition. This circumstance contributes to originating fluctuations in nutritional traits for different cultivation areas and among kernels as well. A full description of the biological mechanisms playing roles in the nutritional quality development of rice kernels is yet the object of scientific investigations [49,50,51]. However, analytical methods of multi-omics have already been applied to complement genomic approaches in targeting quality-related molecules, such as starch, proteins, and secondary metabolites, with respect to their nutritional and medicinal functions [52,53,54,55]. In this study, we confirmed that the nutritional quality of different batches from a single (pedigreed) rice cultivar (Koshihikari), with nominally genetic homogeneity but different geographical characteristics, is indeed inhomogeneous. Moreover, as an original contribution, we also discovered that the most marked inhomogeneities in carbohydrates, proteins, and aromatic amino acids actually existed among rice kernels within the same genomically and geographically homogeneous batches. This finding suggested that rice eating and nutritional qualities could be tailored by simply grouping rice kernels with selected dietetic characteristics from commercially available rice products.

Variations in the metabolite concentrations among rice batches of the same genomic species indeed depend on cultural conditions [56] and decrease with advancing grain development [49,57]. According to Chen et al. [57], the widest separation among cultivars occurs during time intervals corresponding to grain filling and storage compound accumulation. Those researchers observed drastic changes in both metabolic type and the contents of starch carbohydrates, amino acids, and their derivatives (i.e., proteins) during the growth process, which, in turn, greatly differentiated eating and nutritional qualities.

Rice starch consists of amylose and amylopectin, and represents the photosynthetic end product and energy reserve in rice grain [58]. The amylopectin-to-amylose ratio is considered to be the most important factor affecting rice eating quality, [59] because the digestion of starch through enzymatic reactions represents a fundamental process in human nutrition. The rapid digestion of starch occurs in the upper gut and lies at the origin of a rapid release of glucose rings into the bloodstream. This process represents a major trigger of physiological responses, ultimately leading to diabetes, cardiovascular diseases, and some types of cancer [60]. Conversely, the so-called “resistant starch”, namely starch with compositions reaching (largely undigested) the colon, not only enables better glucose control, but also triggers prebiotic effects that allows the development of beneficial colonic microflora [61,62]. Several studies have succeeded in relating the amylose content of white rice kernels to the postprandial glycemic index [63,64,65]. A direct linear relationship was systematically found between the amylopectin fractions in starch and glycemic index values, and the higher the amylopectin content, the higher the glycemic impact of the rice. Accordingly, the systematic availability of rice samples with slow-digesting characteristics (i.e., containing starch high in amylose) is of fundamental importance for diabetic patients. Such rice samples could be key in both curative and preventive nutritional healthcare.

Amino acids are primary molecules in the synthesis of storage proteins in rice kernels, and are precursors for secondary metabolite biosynthesis and energy sources [66]. Although protein content negatively correlates with parameters of rice eating quality, [32,50] proteins represent an important nutrient in rice grains. Early in the seventies, Taira [56] located two main factors that are decisive for the final amount of proteins in genetically homogeneous rice kernels, namely, nitrogen application and irrigation. It has long been known that nitrogen application after heading increases protein content up to ~10% in comparison to its non-application [67]. A continuous nitrogen supply, especially late in grain maturity, promotes the formation of orderly packed starch granules and protein bodies [68]. Such structural variation not only contributes to rice grain appearance and eating qualities, but also affects the secondary structure of proteins and, thus, alters rice digestibility. Regarding the above-mentioned irrigation factor, upland cultures of genetically homogeneous rice systematically manifest protein contents up to 40% higher than those from lowland cultures [69]. Another factor greatly influencing the protein content of rice kernels is the maturity of rice, with immature rice containing more proteins than fully ripened rice [70]. The full set of available data indicates that a high protein content in a rice kernel is indeed not hereditary, but mainly due to cultural conditions, irrigation, and maturity. While the above trends point to the possibility of exploiting changes in metabolites to tailor rice eating and nutritional quality, it is not surprising that such a variety of extrinsic factors could involve broad distributions in protein content and secondary structures, as observed here among nominally homogeneous rice kernels.

The interconversion and accumulation of rice kernel proteins with a specific secondary structure involve crucial healthcare implications. Fractions of specific protein secondary structures are known to belong to specific rice proteins. A circular dichroism study by Mawal et al. [71] on purified rice proteins showed that rice albumin is predominantly composed of β-sheet and β-turn (Type II) structures, while no other rice protein possesses such a peculiar structure. Conversely, the secondary structure of rice prolamin is predominantly α-helical, while glutelin mainly exhibits the random coil structure, accompanied by only a very minor fraction of α-helix. Since random coil and α-helix relate to the highest and lowest protein digestibility, respectively, the fractional amounts of protein secondary structures give a fingerprint of protein digestibility for a given overall content of grain proteins. Note that glutelin is by far the major protein contained in rice kernels (~80%), and prolamin a minor one (5~20%), the remaining fraction being albumin [72]. In other words, prolamin is an indigestible component (i.e., rich in α-helix) which performs as a resistant protein in the digestive tract; it has also been reported to lower blood cholesterol [73]. Therefore, rice kernels low in glutelin and high in prolamin should possess a low protein digestibility and should be preferred for patients with chronic kidney disease [74]. Chronic kidney disease leads, in the long term, to kidney failure and requires hemodialysis or renal transplantation. Notably, a significant fraction (20~40%) of diabetic patients were found to develop chronic kidney disease as a late complication [75,76,77]. This situation requires adequate interventions with low-protein diets [78,79]. Several methods have been proposed for producing such ad hoc rice quality, which include protein extraction via polishing/fermentation [32] or bacteria-derived enzymatic reactions [80]. However, all these treatments negatively impact on the taste, smell, and texture of the rice [81]. In a context of mixed pathologies (i.e., diabetes and chronic kidney disease), a dietetic rice brand offering a blend of kernels low in amylopectin content in an appropriate balance with kernels low in protein and high in α-helix could help with controlling all pathological requirements while yet preserving taste and texture (i.e., without using any low-protein processing procedures). The data presented in the present paper show that this could be possible by using, for example, the low-amylopectin and high-α-helix edges of the Koshihikari Mie sample statistical distributions, as shown in Figure 6 and Figure 7, or other possible combinations. In rice-eating countries, a reduction in rice protein content could allow to insert into the diet a corresponding amount of meat, fish, or soy protein, thus enriching the nutritional value of the diet even under protein restriction [82,83]. Finally, it should also be noted that the dualism glutelin/prolamin also plays an important role in rice antioxidant capacity, the distinct digestibility levels of these protein components being a critical factor in modifying their free radical scavenging activity; the higher the digestibility, the higher the scavenging activity [84].

Phenylalanine and tryptophan amino acids represent fundamental building blocks for proteins and important precursor molecules in secondary metabolism. The abundance of these two aromatic amino acids arises from a specific metabolic pathway referred to as glutamic-oxalacetic transaminease [85,86]. However, they cannot be synthesized in the human body, with their supply only depending on nutritional provision. For this reason, the presence of these essential amino acids in rice kernels should be considered as an important nutritional characteristic for rice brands, including also their antioxidant properties. Moreover, the abundance of phenylalanine and tryptophan free amino acids also represents an important compositional characteristic related to the taste of rice. The contents of tryptophan and phenylalanine were also found to be positively correlated with sensory taste in general [86]. For all these reasons, researchers have looked for modified biosynthetic pathways in order to accumulate selected amino acids in rice at high concentrations [6,87,88]. The present Raman approach could allow for the separation of fractions of rice kernels up to ~8%, which contain phenylalanine and tryptophan fractions in the wt.% intervals 8~10 and 0.8~1.0, respectively (Figure 8).

**Figure 11:**
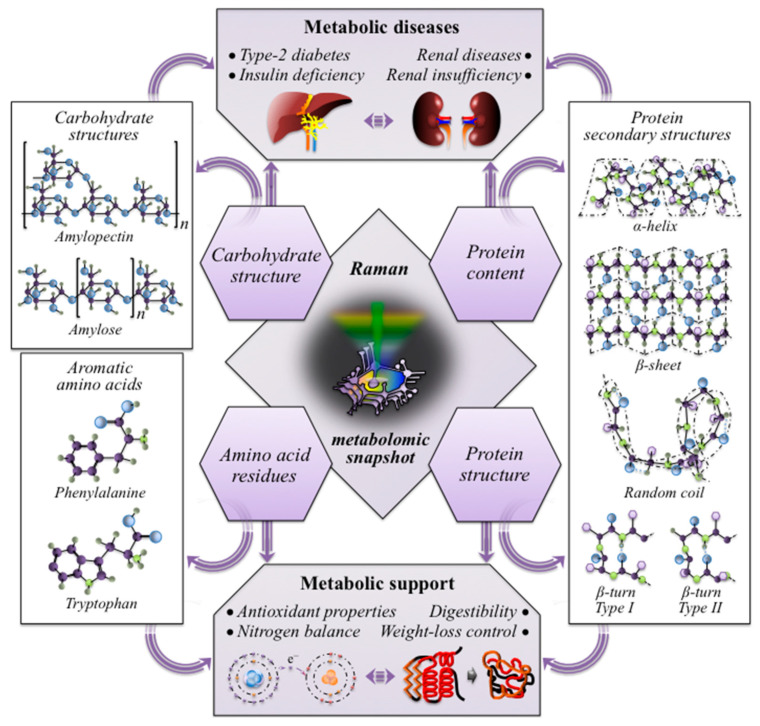
Schematic draft summarizing and linking the impact of rice nutritional traits, as determined by a non-destructive Raman analyses, on human health.

The above-discussed multi-omic concepts regarding the impact of rice nutritional traits on human health, as determined by a non-destructive Raman analysis, are schematically depicted and linked in the schematic draft of Figure 11.

## 5. Conclusions

In this study, Raman spectroscopy emerged as a unique spectroscopic method for prompt, non-contact, and non-destructive analyses of the nutritional traits in individual rice grains. The application of quantitative Raman algorithms to a one-shot non-destructively recorded spectrum makes the automatic screening of a large number of rice kernels practical and economically feasible. Raman technology offers exciting opportunities for enhancing food safety and quality, as well as for systematically controlling health-related issues. As a proof of concept, four main nutritional parameters were selected and the statistical distributions of their fractions were determined from a single-shot Raman spectrum non-destructively recorded over a sample of 300 rice kernels for each of three samples of Koshihikari rice cultivar from different regions of Japan. The selected parameters were as follows: (i) the amylopectin fraction in starch, which directly relates to the postprandial glycemic impact; (ii) the weight fraction of proteins (given in %) as the nutritional counterpart of the carbohydrate content; (iii) the secondary structure of proteins, namely, random coil vs. α-helix fractions as a measure of protein digestibility; and, (iv) the cumulative weight fraction of aromatic phenylalanine and tryptophan amino acids as a measure of protein quality and superior taste.

The possibility of the molecular monitoring of such key nutritional issues at the level of individual rice kernels opens the way to a new approach of rice “dietary engineering” for modern healthcare control without compromising the taste of rice. Future developments of quantitative Raman algorithms could also be possible for additional metabolites, which were spotted in the collected Raman spectra (e.g., β-carotene), upon exploiting the many additional details buried in the Raman spectrum of the rice. Linking the molecular structure of individual rice kernels to glycemic, dietary, and antioxidative characteristics greatly extends the potentiality of medicinal food chemistry, bringing it closer to wide and economically feasible practical applications.

## Figures and Tables

**Table 1:** Critical Q values from the comparisons of studentized range distributions of amylopectin, protein fraction, α-helix, β-sheets, random coils, phenylalanine, and tryptophan (Equation (1)). Abbreviations K, H, and M stand for Kyoto, Hyogo, and Mie. The relationships Kyoto with respect to Hyogo, Kyoto with respect to Mie, and Hyogo with respect to Mie were analyzed (the listed Q values). Values reported in red color represent significant results. The values for Q_0.05_ and Q_0.01_ were 3.32 and 4.13, respectively.

Molecules	K vs. H	K vs. M	H vs. M
Amylopectin	3.24	6.05	2.82
Protein fraction	2.27	14.47	12.20
α-helix	2.77	3.94	1.17
β-sheets	1.70	1.30	0.40
Random coil	1.72	0.02	1.70
Phenylalanine	0.69	12.13	12.82
Tryptophan	3.23	9.32	12.56

## Data Availability

The data used to support the findings of this study can be made available by the corresponding author upon request.
